# Marital status and survival in patients with rectal cancer: A population-based STROBE cohort study: Erratum

**DOI:** 10.1097/MD.0000000000010872

**Published:** 2018-05-18

In the article, “Marital status and survival in patients with rectal cancer: A population-based STROBE cohort study”,^[1]^ which appeared in Volume 97, Issue 18 of *Medicine*, Zhuyue Li and Kang Wang are co-first authors.
